# Therapeutic effects of pentoxifylline on invasive pulmonary aspergillosis in immunosuppressed mice

**DOI:** 10.1186/s12890-021-01396-8

**Published:** 2021-01-19

**Authors:** Chunlai Feng, Ming Zhang, Sujuan Zhang, Jun Zhang, Chong Li, Jun Zhou

**Affiliations:** 1 Department of Respiratory Medicine, The Third Affiliated Hospital of Soochow University, 185 Juqian Street, Changzhou, 213003 China; 2 Comprehensive Laboratory, The Third Affiliated Hospital of Soochow University, Changzhou, China

**Keywords:** Invasive pulmonary aspergillosis, Mouse, Immunosuppression, Pathology, Pentoxifylline, Chitinases, Myeloperoxidase, Interleukin 8

## Abstract

**Background:**

The most common and severe infection of *Aspergillus fumigatus* is invasive pulmonary aspergillosis (IPA), which is usually seen in immunocompromised patients. Neutropenia is the primary risk factor implicated in IPA; however, IPA also occurs in patients without neutropenia, namely, those who are immunosuppressed owing to long-term corticosteroid use. With IPA-associated mortality as high as 51–79%, novel and effective treatment strategies are urgently needed. Pentoxifylline (PTX) has been shown to competitively inhibit the family 18 chitinases in fungi, which may be an new antifungal therapy. Hence, the aim of our study was to compare neutropenic and non-neutropenic IPA mouse models, and to evaluate the effect of PTX on IPA in immunosuppressed mice.

**Methods:**

C57BL/6J mice were pre-treated with cyclophosphamide and hydrocortisone. Neutropenic model IPA mice (CTX-IPA) and non-neutropenic IPA mice (HC-IPA) were established by intranasal administration of *Aspergillus fumigatus* spore suspension. A subset of each group was injected with PTX post-infection. Among these groups, we compared overall survival, pulmonary fungal burden, lung hispathology, and myeloperoxidase (MPO), interleukin 8 (IL-8), and mammalian chitinase concentration in the bronchoalveolar lavage fluid (BALF).

**Results:**

The survival rate of the HC-IPA group was higher than that of the CTX-IPA group, and pulmonary fungal burden was also lower (*p* < 0.05). The CTX-IPA group showed infiltration of alveolae and blood vessels by numerous hyphae of *A. fumigatus*. The HC-IPA group exhibited destruction of bronchi, expansion of alveolar septa, increased macrophages aggregation, significant neutrophil infiltration and a few hyphae in peribronchial areas. After PTX treatment, improvement was observed in survival duration and pulmonary fungal burden in HC-IPA mice. MPO and IL-8 levels were lower in the HC-IPA + PTX group compared to the corresponding levels in the HC-IP group. Chitotriosidase (CHIT1) and Chitinase 3-like 1 (CHI3L1) expression in the HC-IPA group was decreased after PTX treatment (*p* < 0.05).

**Conclusion:**

PTX was found to exert a therapeutic effect in a non-neutropenic mouse model of IPA, which may lead to the development of novel strategies for IPA treatment.

## Background

In recent years, the prevalence of invasive pulmonary aspergillosis (IPA) has increased significantly with an increasing population of immunosuppressed individuals at a high risk of infection [1]. Neutropenia is recognized as the primary risk factor in the occurrence and progression of IPA, and it presents primarily in patients with hematological cancers and those receiving chemotherapy for leukemia. However, an increasing number of recent studies have determined that IPA can also occur in patients without neutropenia, such as those on long-term corticosteroid therapy, and those with chronic obstructive pulmonary disease or solid organ tumors [2, 3]. Recent studies on antifungal drugs have reported considerable progress; however, the problems of low therapeutic efficacy and drug resistance persist. The mortality rate of patients with IPA is as high as 51–79% [4, 5]; treatment is prolonged, expensive, and is a serious disease burden on society and individuals. Thus, more effective drugs and procedures are urgently needed for the treatment of IPA.

Chitinases are chitin-degrading enzymes, present in a wide range of organisms, such as insect, fungi, plants and animals including mammals, and have important biophysiological functions. Fungal chitinase plays an important role in the growth, nutrition and development of fungi [6, 7]. Research shows that disruption of the chitinase gene from Aspergillus nidulans decreases germination frequency and hyphal growth [8]. In Candida albicans, deletions in the chitinase genes inhibit cell separation [9]. Additionally, chitinases are essential for sexual development in in Cryptococcus neoformans [10]. So, it indicates the potential utility of chitinases as targets for the development of antifungal agents. As there is no chitin in the human body, targeting its metabolism will be innocuous to mammals. Therefore, inhibitors that target chitinase are good choices for use as antifungal agents. As a methylxanthine derivative, pentoxifylline (PTX) was originally used primarily for the treatment of vascular dysfunction. Additionally, PTX exerts cellular effects on polymorphonuclear neutrophils (PMNs), inhibits inflammatory cytokines, and reverses the effects of these cytokines on phagocytes, such as leukocyte adherence, migration, and degranulation [11]. The therapeutic use of PTX has been studied in different conditions, including Sepsis syndrome, shock, and acute lung injury (ALI) [12–14]. In recent years, its function as a chitinase inhibitor has been characterized [15–17]. In a study by Fancesco et al. [15], it was found that methylxanthine derivatives such as caffeine and PTX inhibit chitinase. PTX has a stronger inhibitory effect on family 18 chitinases, with a Ki of 37 µM against *Aspergillus fumigatus* chitinase, and may also serve as a potential anti-aspergillus drug. In vitro, fungal tests have shown that PTX can inhibit the growth of Cryptococcus neoformans and Aspergillus, which suggests that PTX possesses antifungal properties related to its inhibition of fungal chitinases [18]. However, the effect of PTX on IPA has not been well-reported. In the present study, we developed and compared IPA models in mice with or without neutropenia, observed the therapeutic effect of PTX against IPA in mice with varying immunosuppression status, and explored the potential molecular mechanisms associated with treatment. Here, we report that PTX exerts a therapeutic effect in a non-neutropenic mouse model of IPA, which could potentially be developed as a new treatment approach. The characteristics of “old medicine new use” can be reflected on PTX.

## Methods

### Fungal strain

A clinical isolate of *A. fumigatus* (AF001) obtained from patients with proven IPA in Jinling Hospital (Nanjing, China), was used in the present study. The clinical isolate has been identified as *A. fumigatus* by macroscopic and micromorphological characteristics, thermotolerance at 48 °C, and molecular identification in the Microbiological Laboratory of Jinling Hospital (Nanjing, China). And the antifungal susceptibility testing in vitro was performed by broth microdilution according to the European Committee on Antimicrobial Susceptibility Testing (EUCAST) reference method. The information has been reported in other publication [19]. In addition, the strain has been used by one of us (Ming Zhang) to induce experimental animal IPA [20]. The cells were inoculated on Sabouraud dextrose agar (SDA) medium and cultured for 7 days at 37 °C. *A. fumigatus* spores were collected in 10 mL phosphate buffered solution (PBST) (containing 0.1% Tween 80), and subsequently filtered through four layers of gauze to eliminate hyphae and residual medium. The spores were counted using a hematocytometer, the concentration was adjusted to 1 × 10^8^ conidia/mL, following which they were stored at 4 °C for use within 72 h. The viability of the spore was assessed by tenfold serial dilutions plating.

### Experimental animals

A total of 120 clean-grade, healthy C57BL/6J female mice aged 6 weeks old and with a mean body weight of 20 g (± 2 g) were used. The animals were housed under standard conditions and allowed access to food and water ad libitum. Cages were kept in the animal laboratory of Guangzhou Yuansheng Medical Technology Co., Ltd. The animal laboratory approval number is SYXK (shiyanxuke) (Guangzhou) 2015-0150. This study was performed according to the National Institutes of Health Guide for Care and Use of Laboratory Animals.

### Establishment of A. fumigatus infection models

Neutropenic IPA mouse model (CTX-IPA): To establish an immunosuppressed state, an intraperitoneal injection of 250 mg/kg cyclophosphamide ( Shengdi Pharmaceutical Co., Ltd. Jiangsu,China) was administered 3 days (d-3) and 1 day (d-1) before infection. Blood from the retro-orbital venous plexus was collected for neutrophil count estimation. Non-neutropenic IPA mouse model (HC-IPA): to establish an immunosuppressed state, a subcutaneous injection of 62.5 mg/kg hydrocortisone (Jinyao Pharmaceutical Co., Ltd. Tianjin,China) was administered every other day, from 5 days before infection (d-5) to the day after infection (d + 1), for a total of four doses. The two groups of mice were administered an intranasal instillation of 50 μL (1 × 10^8^ conidia/mL) of *A. fumigatus* spore suspension. We also used control mice that were neither immunosuppressed nor exposed to *A. fumigatus* (control), and a group of immunocompetent mice exposed to *A. fumigatus* as described above (immunocompetent/exposed). 120 animals were randomized into groups of 20 mice. For evaluation of survival, eight mice were included in each group.

### PTX administration and experimental groups

An intraperitoneal injection of PTX (Xi'an High-tech Pharmaceuticals, China. Approval number: National Drug Standard H2004578) (20 mg/kg) was administered on day 1 post-infection and continued daily for 5 days. The neutropenic IPA model mice were divided into two groups, namely a CTX-IPA model group and a pentoxifylline-treated (CTX-IPA + PTX) group. The non-neutropenic IPA model mice were divided into two groups, namely an HC-IPA model group and a pentoxifylline-treated (HC-IPA + PTX) group. Various groups of 20 mice each were selected and randomised.

### Observational indices

The general condition of the mice after infection, including weight, death, and activity, was observed and recorded. All the mice lost more than 25% of their original body weight and were euthanized.

### Determination of pulmonary fungal burden in mice

The right lung was freshly harvested from mice on days 1, 3, and 5 after infection. After weighing, 1 g of wet lung tissue was diluted in 10 mL PBS (containing 0.1% Tween 80), homogenized, and diluted through a tenfold gradient for culture, inoculated in Sabouraud culture medium, and cultured at 37 °C. The colonies were counted after 24 h by a lab assistan who was blinded to the sample numbers. And the fungal burden of the tissue was expressed as log_10_ CFU/g (colony-forming unit) tissue.

### Histopathology

Four mice per group were assigned for histopathological studies. The lung tissues of the mice were harvested, fixed in paraformaldehyde, embedded in paraffin, cut into 4-μm slices. The sections were stained with hematoxylin–eosin (H&E)for tissue examination and with periodic acid–Schiff (PAS) for fungus detection. The pathological changes in IPA mice were estimated by a pathologist who was blinded to allocation groups. Lesions (interstitial and alveolar edema, cellular infiltration, hemorrhage) were graded from 0 (normal), 1 (mild), 2(moderate), to 3 (severe).

### Measurement of inflammatory factors

Mice were sacrificed on days 1 and 3 after infection. Phosphate buffer saline (PBS) (200 μL) was injected into the right lung by tracheal intubation. After a short period of time, the fluid was withdrawn into an Eppendorf test tube and flushed three times. The recovery rate was 80–90%. The recovered fluid was the BALF and was frozen for storage. Enzyme linked immunosorbent assay (ELISA) was used to measure the levels of interleukin 8 (IL-8) and Chitinase 3-like 1 (CHI3L1) (CUSABIO BIOTECH Co. Ltd., Wuhan, China) in the BALF.

### Myeloperoxidase (MPO) assay

MPO activity in the BALF was determined using a commercially available assay kit (Jiancheng Bioengineering Institute, Nanjing, China). All procedures were according to the manufacturer’s instruction. The enzymatic activity was determined spectrophotometrically by measuring the change in absorbance at 460 nm using a 96-well plate reader, and the data was presented as units per litre (U/L).

### Determination of chitotriosidase (CHIT1) activity

Chitotriosidase activity was determined in BALF samples by a spectrofluorometric method according to Hollak et al. [21]. Briefly, 5 μL of BALF was mixed with 100 μL of 22 μmol/L 4-methylumbelliferryl-β-d-*N*-*N′*-*N″*-triacetylchitotriosidase(Sigma Chemical Co., St. Louis, MO) in Mcclvain’s phosphate-citrate buffer; pH = 5.2, for 1 h at 37.0 °C in darkness. The reaction was terminated by the addition of 0.3 mmol/L glycine/sodium hydroxide buffer( pH 10.6). The fluorescence intensity of 4-methylumbelliferone was measured using a fluorophotometer at excitation and emission wave lengths of 358 nm and 448 nm, respectively. CHIT1 activity was calculated by comparing with the standard curve of 4-methylumbelliferone and expressed as nanomoles of substrate hydrolyzed per milliliter per hour (nmol/mL/h).

### Statistical analysis

Student’s *t*-test was used to compare the means between two groups. One-way analysis of variance (ANOVA) was used for comparison among multiple groups of data. Survival curves were plotted using the Kaplan–Meier method. The log-rank test was used for comparison of survival rates. Differences with *p* < 0.05 were considered statistically significant.

## Results

### Comparison of neutrophil counts between two immunosuppressed mouse models of Aspergillus infection

The neutrophil count in the blood from the CTX-IPA group mice was significantly lower than that in the blood from the control group and immunocompetent/exposed group mice (*p* < 0.05). In the HC-IPA group, the peripheral blood neutrophil count was in the normal range after infection [22], and there was no significant difference compared to that in the control group (Table 1).

**Table 1:** Peripheral blood neutrophils from mice infected with *A. fumigatus*

Group	Neutrophils (mean × 10^3^/µL ± SD)^a^	
	Day 1 post-infection	Day 3 post-infection
Control	0.95 ± 0.29	0.88 ± 0.1
Immunocompetent/exposed	0.88 ± 0.19	0.94 ± 0.67
CTX-IPA	0.01 ± 0.01*^,#^	0.02 ± 0.01*^,#^
HC-IPA	1.43 ± 0.77	2.55 ± 1.68

Treatments were as follows: cyclophosphamide (250 mg/kg) intraperitoneally on the day before the 1st and 3rd challenges (CTX-IPA); hydrocortisone (62.5 mg/kg) subcutaneously every other day, from 5 days before infection (d-5) to the day after infection (d + 1)(HC-IPA);

Mice were infected intranasally with 50 μL (1 × 10^8^conidia/mL) of *A. fumigatus* spore suspension. The groups of control received 50 μL of phosphate buffer saline (PBS). Blood samples were collected from four mice per group at the indicated days post-infection

^a^SD, standard deviation; *p < 0.05, compared with the control group. ^#^*p* < 0.05, compared with the immunocompetent/exposed group

### General condition and survival rates of different types of IPA mi

**Fig. 1:**
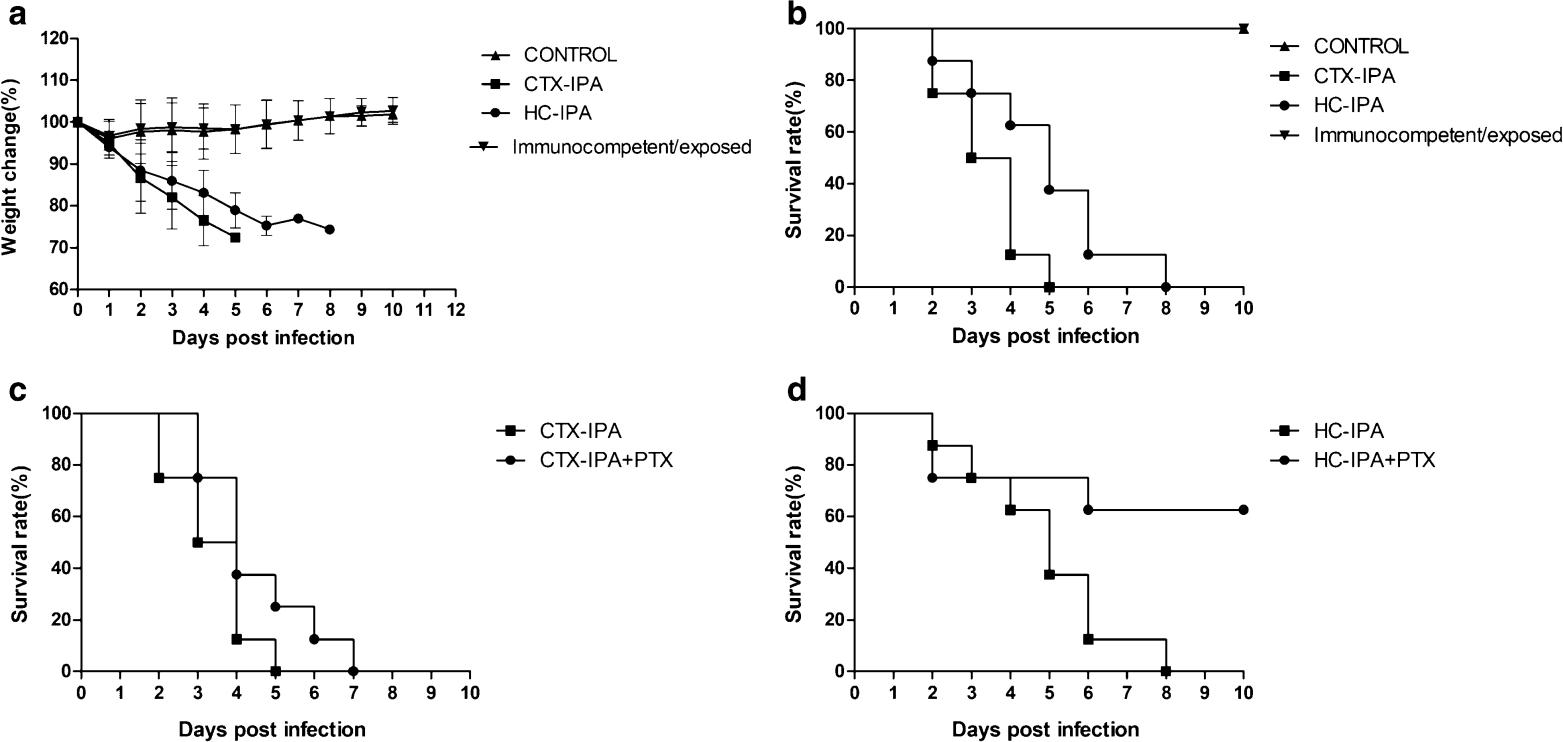
Effect of PTX on the survival of mice with IPA. Immunosuppression of mice infected with *A. fumigatus* was achieved by daily intraperitoneal administration of 20 mg/kg PTX from day 1 post-infection, which was continued for 5 days. **a** Weight change of mice with IPA. **b** Survival rate of different types of IPA mice. **c** Survival rate of CTX-IPA mice after PTX treatment. **d** Survival rate of HC-IPA mice after PTX treatment (n = 8 in each group)

On day 1 after *Aspergillus* infection, the CTX-IPA group mice exhibited decreased appetite, fur thinning, and loss of weight. On day 2, the mice developed shortness of breath, wheezing, and hemiplegia. By day 5 of infection, all the mice lost more than 25% of their original body weight and were euthanized via cervical dislocation. In the HC-IPA group, activity levels decreased, breathing was rapid, and body weight gradually decreased, but there was no evidence of hemiplegia. The median survival of the mice in the CTX-IPA group was significantly lower than that in the HC-IPA group (3 days and 5 days, respectively) (*p* < 0.05) (Fig. 1).

### Lung histopathology in IPA mice with varying immunosuppression status

**Fig. 2:**
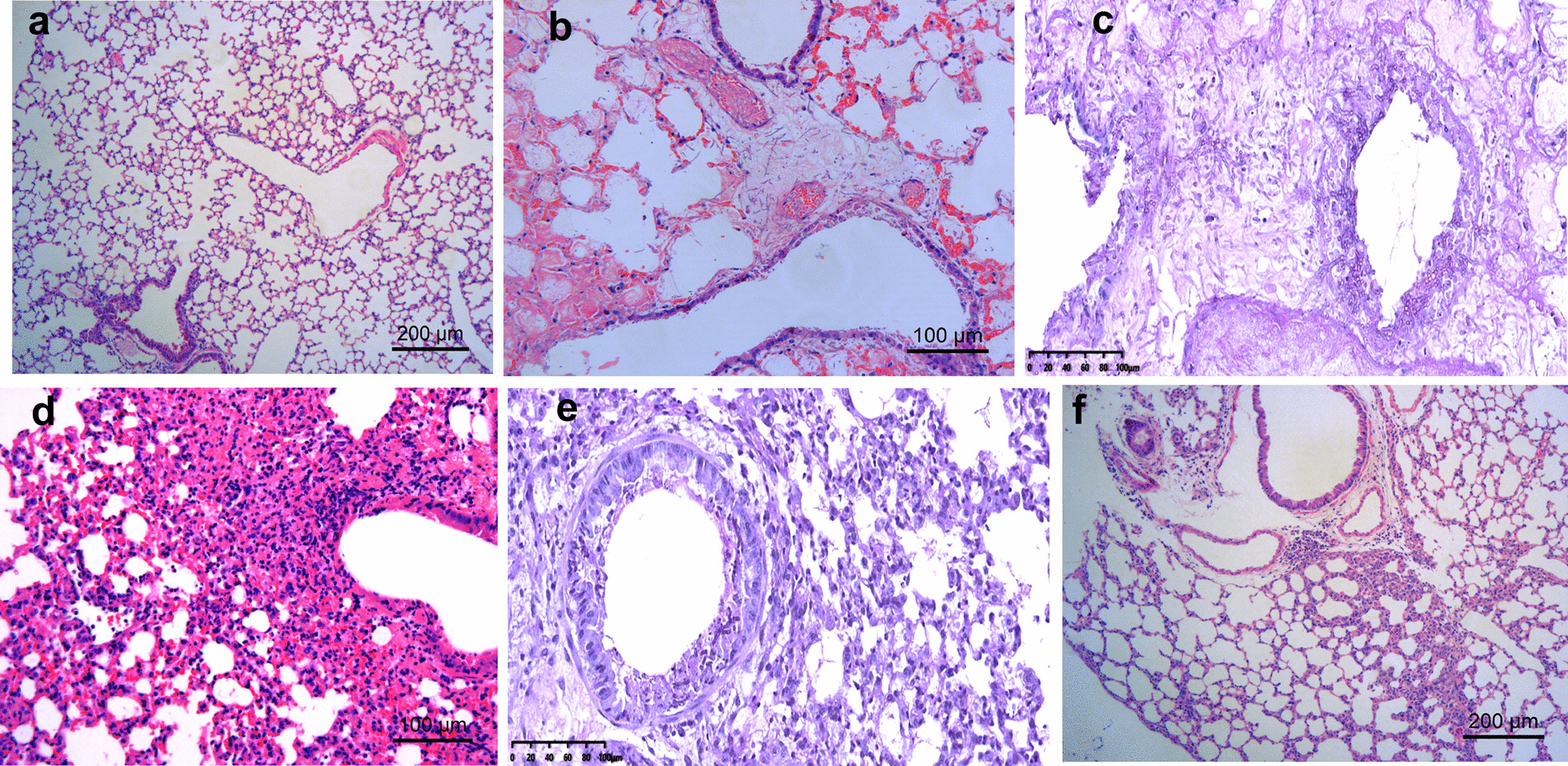
Histopathology of the lungs from mice with IPA on day 3 post-infection. Representative images of HE staining of lung tissue of **a** control mice, showing normal bronchioles and alveoli; **b** CTX-IPA mice showing diffuse pulmonary edema and congestion within alveolae; **c** PAS-stained lung sections of mice with CTX-IPA, showing invasion of bronchi, alveolae and blood vessels by numerous hyphae of *A. fumigatus*; **d** HE stain of lung tissue from HC-IPA mice showing destruction of bronchi, expansion of alveolar septa, increased macrophages aggregation and numerous inflammatory cells, especially neutrophils; **e** PAS-stained lung sections of mice with HC-IPA, showing a small mounts of hyphae in peribronchial areas; **f** HE stain of lung section of HC-IPA mice treated with PTX showing a small number of inflammatory cell in the lung septum, including neutrophils and lymphocytes. For **a** and **f** scale bars = 200 µm. For **b**–**e** scale bars = 100 µm

On day 3 after *Aspergillus* infection, the CTX-IPA group mice exhibited severe pathological changes, including diffuse pulmonary edema, hemorrhagic necrotizing inflammation, and invasion of alveolae and blood vessels by numerous hyphae of *A. fumigatus* also can be seen. The pathological changes in the HC-IPA group were destruction of bronchi, expansion of alveolar septa, macrophages recruitment, a large amount of neutrophil infiltration, increased lymphocyte aggregation, and a few hyphae within peribronchial areas (Fig. 2).

### Fungal burden in IPA mice with varying immunosuppression status

On day 3 after infection, the pulmonary fungal burden in the HC-IPA group was significantly lower than that in the CTX-IPA group (*p* < 0.05). *A. fumigatus* colonies were detected in the brain and kidney of the CTX-IPA group on day 3 post-infection. However, no *A. fumigatus* colony was detected in the brain and kidney of the immunocompetent/exposed and HC-IPA groups (Table 2).

**Table 2:** Fungal burden of organs in mice infected with *A. fumigatus*

Group	Fungal burden of organs(mean log10 CFU/g ± SD)^a^		
	Lung	Brain	Kidneys
Control	None	None	None
Immunocompetent/exposed	None	None	None
CTX-IPA	4.19 ± 0.35*	2.35 ± 0.33	1.60 ± 0.69
HC-IPA	3.27 ± 0.41*	None	None

Mice were inoculated intranasally with 50 μL (1 × 10^8^ conidia/mL) of *A. fumigatus* spore suspension in IPA group and immunocompetent/exposed group. The groups of control received 50 μL of phosphate buffer saline (PBS). Tissue samples were collected from four mice per group on day 3 post-infection. Fungal burden in mice was measured 3 day after infection. Fungal burden was expressed as log10 CFU/g of tissue

^a^CFU, colony-forming unit; SD, standard deviation; *p < 0.05, CTX-IPA group versus HC-IPA group

### Changes in survival rate of IPA model mice after PTX treatment

The survival rate of mice in the CTX-IPA group was not significantly different after PTX treatment (Fig. 1c).The survival rate of mice in the HC-IPA group was 62.5% 10 days after treatment with PTX, which was significantly higher than that in mice not treated with PTX (*p* < 0.05) (Fig. 1d).

### Changes in pulmonary fungal burden after PTX treatment of IPA model mice

The pulmonary fungal burden in the CTX-IPA + PTX group decreased significantly on day 3 after infection compared to the corresponding parameter in the CTX-IPA group (*p* < 0.05). No *A. fumigatus* colony was detected in the lungs of the HC-IPA + PTX group on days 3 and 5 post-infection (Table 3).

**Table 3:** Effect of PTX on pulmonary fungal burden

Group	Pulmonary fungal burden (mean log10 CFU/g ± SD)^a^		
	Day 1 post-infection	Day 3 post-infection	Day 5 post-infection
Immunocompetent/exposed	5.1 ± 0.43^c^	None	None
CTX-IPA	6.18 ± 0.41^c^	4.19 ± 0.35*	ND^b^
CTX-IPA + PTX	5.61 ± 0.52	2.77 ± 1.06*	ND^b^
HC-IPA	5.95 ± 0.65^c^	3.27 ± 0.41	2.34 ± 0.24
HC-IPA + PTX	5.13 ± 1.30	None	None

Immunosuppressed mice, infected with *A. fumigatus,* were injected intraperitoneally with 20 mg/kg PTX daily for 5 days. Lung tissue samples from four mice per group were collected on days 1, 3, and 5 post-infection

^a^CFU, colony-forming unit; SD, standard deviation

^b^ND, not detected

^c^*p* < 0.05 compared with the immunocompetent/exposed group

*p < 0.05, CTX-IPA group vs. CTX-IPA + PTX group

### MPO activity

**Fig. 3:**
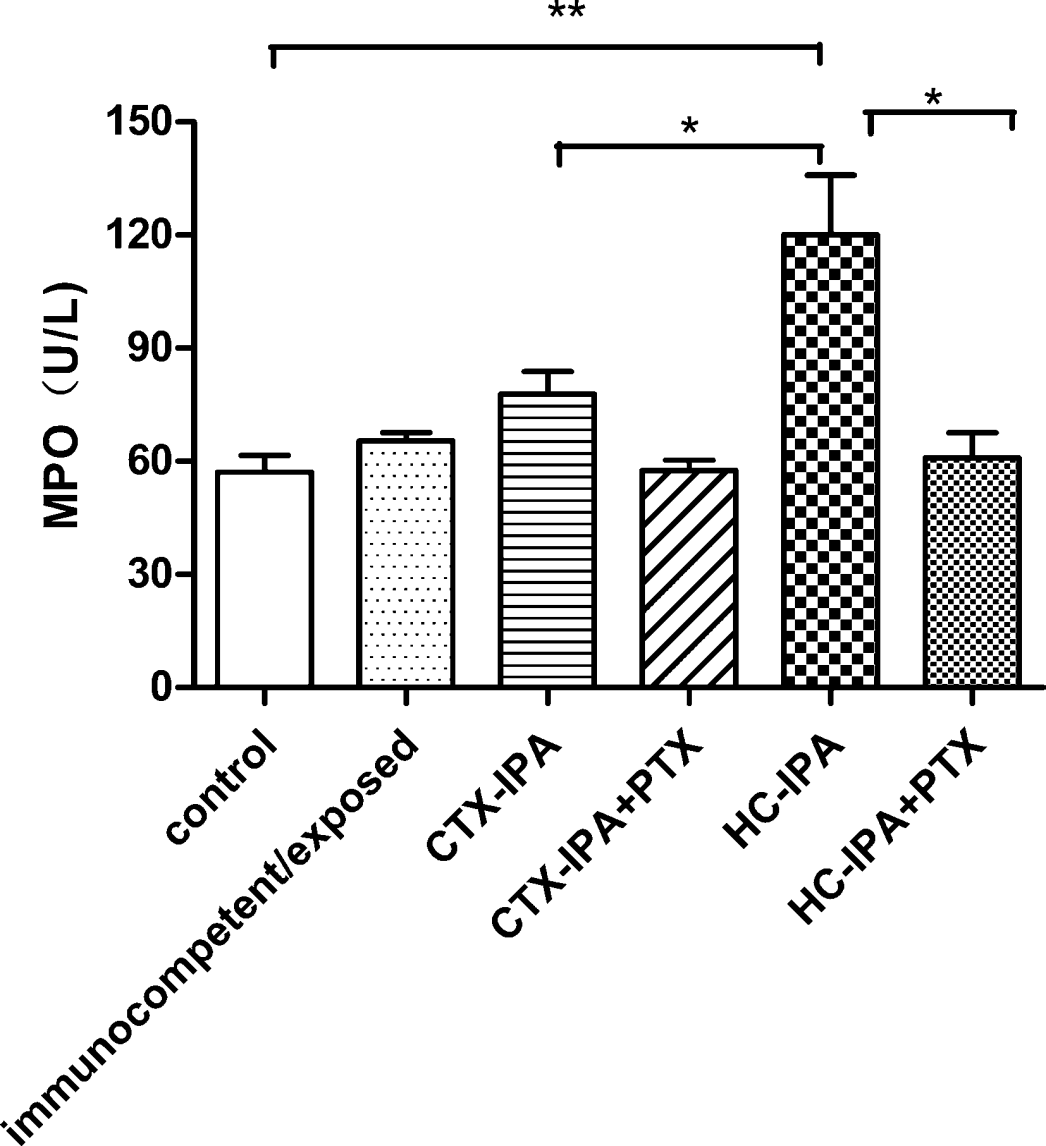
MPO activity in the BALF from mice with IPA. BALF samples were collected from four mice per group on day 3 post-infection. Data are expressed as mean ± SEM (Standard Error of Mean); **p < 0.01, compared with the activity level in the control group. *p < 0.05 for HC-IPA group vs. CTX-IPA group. HC-IPA group vs. HC-IPA + PTX group (n = 4 in each group)

In HC-IPA mice, MPO activity was higher than that in the control group, immunocompetent/exposed group, and CTX-IPA group (p < 0.05) on day 3 after infection After PTX treatment, MPO activity in the BALF of HC-IPA + PTX group decreased significantly (*p* < 0.05) (Fig. 3).

### Effect of PTX on IL-8 in the BALF of IPA model mice

**Fig. 4:**
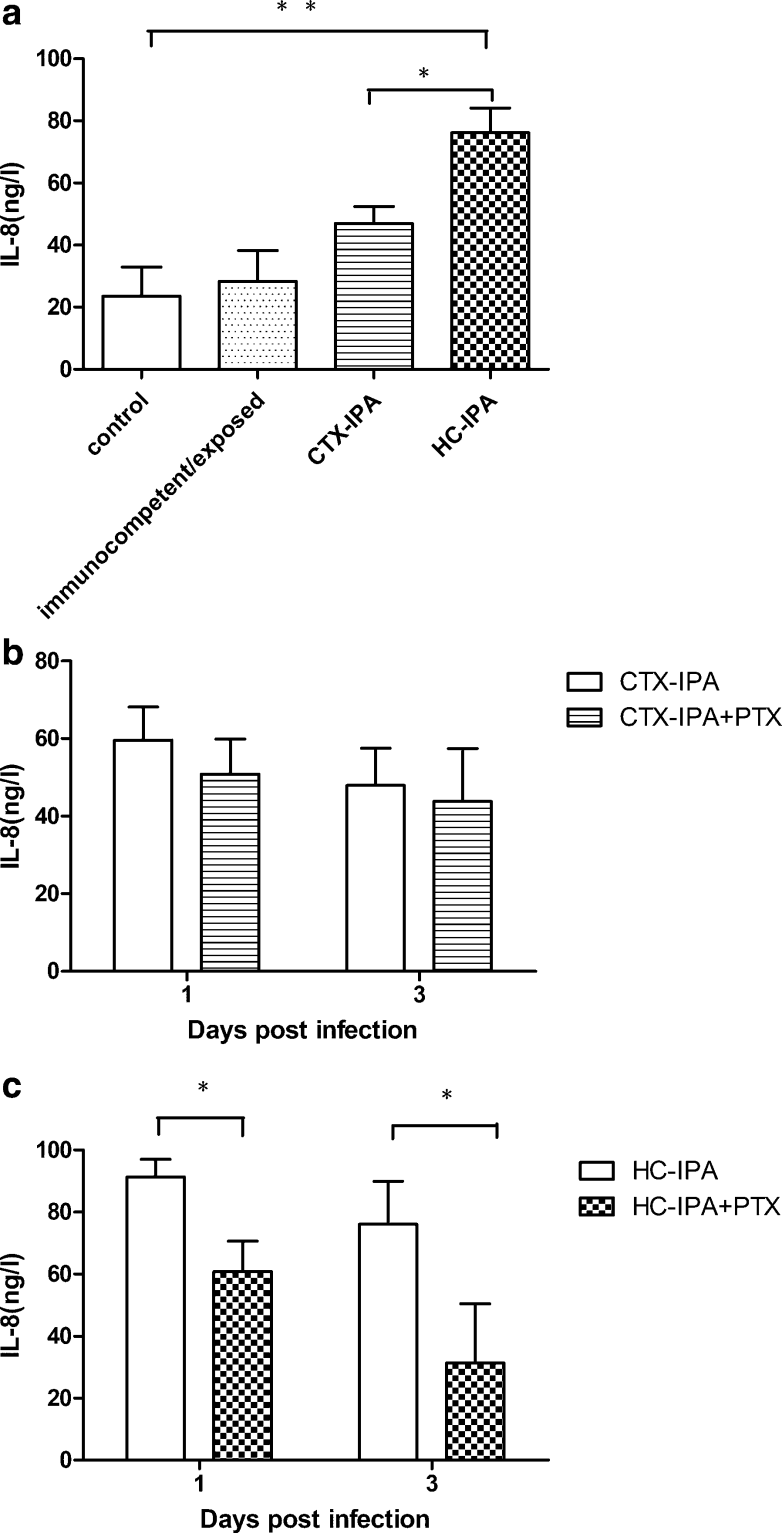
Effect of PTX on IL-8 levels in the BALF from mice infected with *A. fumigatus. a* IL-8 expression in the BALF from mice with IPA on day 3 post-infection. **p < 0.01 for HC-IPA vs. control group or immunocompetent/exposed group. *p < 0.05 for HC-IPA group vs. CTX-IPA group; **b** IL-8 expression in the BALF from CTX-IPA mice after PTX treatment on days 1 and 3 post-infection. **c** IL-8 expression in the BALF from HC-IPA mice after PTX treatment on days 1 and 3 post-infection. *p < 0.05 for HC-IPA group vs. HC-IPA + PTX group (n = 3 in each group).

On day 3 after infection, IL-8 levels in the BALF of the CTX-IPA and HC-IPA mice were higher than those in the control group and the immunocompetent/exposed group. The difference between the HC-IPA group and the control group was statistically significant (*p* < 0.01). IL-8 levels in the HC-IPA group were higher than those in the CTX-IPA group (*p* < 0.05) (Fig. 4a). IL-8 production in the HC-IPA + PTX group on days 1 and 3 after infection (60.82 ± 9.80 and 31.39 ± 19.08 ng/L, respectively) was significantly lower than that in the HC-IPA group (91.40 ± 5.68, 76.13 ± 13.82 ng/L, respectively, *p* < 0.05) (Fig. 4c).

### Effect of PTX on CHIT1 and CHI3L1 in IPA model mice

**Fig. 5:**
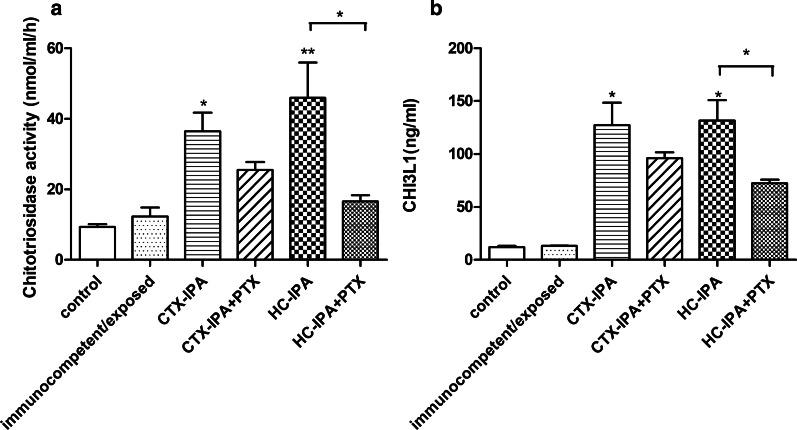
Effect of PTX on CHIT1 activity and CHI3L1 in the BALF from mice with IPA. BALF samples were collected on day 3 post-infection. **a** Inhibition of CHIT1 activity in BALF samples from mice with IPA after PTX treatment. *p < 0.05, **p < 0.01, compared with the activity level in the control group. *p < 0.05, HC-IPA group vs. HC-IPA + PTX group. **b** Effect of PTX on CHI3L1 levels in the BALF from mice with IPA. *p < 0.05, compared with the corresponding levels in the control group. *p < 0.05, HC-IPA group vs. HC-IPA + PTX group (n = 4 in each group)

CHIT1 activity in the BALF from the CTX-IPA and HC-IPA groups was significantly higher than in the BALF from the control group (*p* < 0.05) (Fig. 5a). After PTX treatment, CHIT1 activity decreased in both types of IPA mice; however, CHIT1 activity in the HC-IPA + PTX group was significantly lower than that in the HC-IPA group (*p* < 0.05) (Fig. 5a). On day 3 after infection, the BALF of the CTX-IPA and HC-IPA groups had high levels of CHI3L1 (vs. the control group,* p* < 0.05) (Fig. 5b). After PTX treatment, CHI3L1 levels decreased in the HC-IPA group (*p* < 0.05) (Fig. 5b).

## Discussion

In the present study, a neutropenic IPA mouse model (CTX-IPA) was primarily characterized by the invasion of alveolae and blood vessels by numerous hyphae of *A. fumigatus*. Systemic fungal dissemination also occurred in the CTX-IPA group. In contrast, for the non-neutropenic IPA mouse model (HC-IPA), we observed only a few hyphae and massive neutrophil aggregation within pulmonary tissue lesions. Compared to the CTX-IPA group mice, the HC-IPA group mice survived longer, and had lower pulmonary fungal burden. Our data are consistent with previous animal model studies [23, 24], and elucidated the clinical characteristics of IPA [25, 26]. Specifically, in patients with neutropenia, aspergillosis affects the blood vessels, the frequency of systemic dissemination is high, and the clinical progression is rapid. Alternatively, *A. fumigatus* in non-neutropenic patients does not involve the blood vessels, the inflammatory reactions in the lungs are severe, and the clinical course of the disease is longer and progresses relatively slowly. Taken together, these data suggest that for patients with IPA, and in animal models of IPA, the clinical characteristics, response to therapy, and prognosis differ according to the immune status of the host. Therefore, the immune and inflammatory response to *A. fumigatus* could be modulated for therapeutic benefit [27].

Currently, antifungal drugs remain the principal treatment modality for IPA. PTX has been shown to competitively inhibit the family 18 chitinases in fungi, which could potentially be developed as a new antifungal therapy. In a murine model of cerebral cryptococcosis, PTX in combination with amphotericin B improved survival, decreasing the fungal burden [28]. Lopera et al*.* [29] found that PTX reduces the pulmonary fungal burden in mice with pulmonary paracoccidioidomycosis, pulmonary inflammation, and the extent of pulmonary fibrosis. Our finding showed that PTX treatment significantly reduced pulmonary fungal burden in both the CTX-IPA and HC-IPA mouse models (albeit to a greater extent in the HC-IPA group). PTX significantly improved the survival rate of mice in the HC-IPA group, but failed to improve the survival rate of CTX-IPA mice. This may be related to the high pulmonary fungal burden in CTX-IPA and fungal dissemination to other organs such as the brain.

In addition to antifungal properties, PTX exerts strong anti-inflammatory and immunomodulatory effects. PTX can inhibit neutrophil activation, migration, and adhesion [11, 30], in addition to its inhibitory effect on inflammatory cytokines [31, 32].

To further investigate the therapeutic effects of PTX on IPA mice and its related mechanisms, we measured MPO activity and IL-8 levels in IPA mice. MPO activity was used as an index to evaluate the accumulation of neutrophils in tissues [33]. IL-8 is a cytokine that can mediate chemotaxis and activate neutrophils, and the proteinases released by neutrophils in inflamed tissues enhance the activity of IL-8 [34]. In the present study, MPO activity and IL-8 levels in the BALF from the HC-IPA group were higher than the corresponding parameters in the control group, immunocompetent/exposed group, and the CTX-IPA group, which is consist with the pathological changes seen in the lungs of the mice. However, CTX-IPA mice still exhibited MPO activity which could be due to residual MPO activity from monocytes [24]. In the HC-IPA group, numerous neutrophils aggregated in the lungs of the mice, and the local inflammatory reaction in the lungs was more pronounced than that in the CTX-IPA mice. PMNs are essential for defense against fungal infection. A variety of enzymes of PMNs such as proteolytic enzymes, lysozyme, and myeloperoxidase are released into the phagosome to destroy microorganisms. However, the excessive release of oxidants and proteases may result in organ injury [11, 35]. Certain studies [25, 36] have found that non-neutropenic IPA lung injury is not caused by *Aspergillus* invasion, but by large numbers of neutrophils migrating to the site of infection and participating in the inflammatory response. We found that MPO and IL-8 levels in the BALF of HC-IPA mice decrease significantly after PTX treatment. Our data suggest that PTX may reduce the lung inflammatory response in HC-IPA mice by attenuating neutrophil activation and inhibiting inflammatory cytokines such as IL-8, thereby increasing the survival rate.

In addition to *A. fumigatus* chitinase, the activity of mammalian chitinases (CHIT1) and acidic mammalian chitinase (AMCase) is inhibited by PTX [15]. CHIT1 and AMCase are members of the 18-glycosyl-hydrolase family, together with chitinase-like proteins that lack enzymatic activity. The recent findings demonstrate that CHIT1 not only catalyze the hydrolysis of chitin to fight against chitin-containing human pathogens, but also play crucial role in the immune response and in disease states where inflammatory responses prevail [37, 38]. The high expression of CHIT-1 was found variably modulated in classical activated macrophages (M1) and alternative activated macrophages (M2) which supporting that CHIT1 was mediator of innate and acquired immunity [39, 40]. However, increased secretion of CHIT-1 can also be damaging to host tissues. A study demonstrates that chitin recognition via CHIT1 promotes harmful T-helper type 2 (Th2) response to cryptococcal infection. The finding suggests that treatments aimed to suppress the detrimental Th2 response by inhibiting CHIT1 maybe necessary to improve progression of Cryptococcosis [41]. In the study, PTX treatment significantly decreased CHIT1 activity in murine BALF from HC-IPA group the survival rate of which is increased. Our finding suggest that PTX may suppress host immune response and improved outcome of HC-IPA by restraining CHIT1 activity. Likewise, CHI3L-1 are widely involved in the human immune inflammatory response. CHI3L1 activates the Protein Kinase B(AKT) and phosphoinositide-3 kinase signaling pathway, which is associated with various disorders such as pneumonia and inflammatory bowel disease [42]. Some studies [17, 43] have found that methylxanthine derivatives such as PTX and caffeine can inhibit intestinal inflammation and treat chronic intestinal inflammatory diseases by inhibiting the secretion of CHI3L1 by human colon epithelial cells. Our finding also show that PTX treatment significantly decreased CHI3L1 levels from HC-IPA group. Therefore, we propose that PTX may reduce the lung inflammatory response and improve progression in HC-IPA mice by inhibiting CHIT1 and CHI3L1. Further studies on the exact mechanism involved are required.

However, our study had certain limitations. First, we did not design a control group of mocking PTX administration. Second, the comparison of results obtained with other antifungal drugs was lacking. Third, there is no detailed study on how PTX inhibits lung inflammation in HC-IPA by inhibiting chitinase. It is impossible to distinguish whether the anti-chitinase activity of PTX is due to the inhibition of host or fungal chitinases. Further studies to separate the effects of PTX on host and fungal chitinases are required.

Since azoles and amphotericin B are classic antifungal agents [44], in future studies, we expect to compare the therapeutic effects of PTX with the effects produced in HC-IPA mice, and conduct further in vitro studies to characterize in detail the mechanisms by which PTX inhibits chitinase.

## Conclusion

The present study compared the therapeutic effects of PTX in IPA models of mice with different levels of immunocompetence and found that PTX has a therapeutic effect in non-neutropenic mice. The associated molecular mechanism may be related to chitinase inhibition, thus aiding in the development of novel therapeutic options for IPA.

## Data Availability

The datasets used and/or analysed during the current study are available from the corresponding author on reasonable request.

## Abbreviations

BALF: Bronchoalveolar lavage fluid
IPA: Invasive pulmonary aspergillosis
PTX: Pentoxifylline
IL-8: Interleukin 8
MPO: Myeloperoxidase
CHIT1: Chitotriosidase
CHI3L1: Chitinase 3-like 1
CTX-IPA: Neutropenic model IPA mice
HC-IPA: Non-neutropenic IPA mice
PMNs: Polymorphonuclear neutrophils
ALI: Acute lung injury
SDA: Sabouraud dextrose agar
PBST: Phosphate buffered solution
SYXK: Shiyanxuke
CTX-IPA + PTX: Pentoxifylline-treated Neutropenic model IPA mice
HC-IPA + PTX: Pentoxifylline-treated non-neutropenic IPA mice
CFU: Colony-forming unit
HE: Hematoxylin–eosin
PAS: Periodic acid–Schiff
PBS: Phosphate buffer saline
ELISA: Enzyme linked immunosorbent assay
ANOVA: Analysis of variance
SD: Standard deviation
ND: Not detected
AMCase: Acidic mammalian chitinase
EUCAST: Broth microdilution according to the European Committee on Antimicrobial Susceptibility Testing
SEM: Standard error of mean
AKT: Protein Kinase B
